# Recent Advances of Curcumin in the Prevention and Treatment of Renal Fibrosis

**DOI:** 10.1155/2017/2418671

**Published:** 2017-05-04

**Authors:** Xuejiao Sun, Yi Liu, Cheng Li, Xiting Wang, Ruyuan Zhu, Chenyue Liu, Haixia Liu, Lili Wang, Rufeng Ma, Min Fu, Dongwei Zhang, Yu Li

**Affiliations:** ^1^Preclinical Medicine School, Beijing University of Chinese Medicine, Beijing 100029, China; ^2^Chinese Material Medical School, Beijing University of Chinese Medicine, Beijing 100029, China; ^3^The Research Institute of McGill University Health Center, Montreal, QC, Canada H4A 3J1; ^4^Diabetes Research Center, Beijing University of Chinese Medicine, Beijing 100029, China

## Abstract

Curcumin, a polyphenol derived from the turmeric, has received attention as a potential treatment for renal fibrosis primarily because it is a relatively safe and inexpensive compound that contributes to kidney health. Here, we review the literatures on the applications of curcumin in resolving renal fibrosis in animal models and summarize the mechanisms of curcumin and its analogs (C66 and (1E,4E)-1,5-bis(2-bromophenyl) penta-1,4-dien-3-one(B06)) in preventing inflammatory molecules release and reducing the deposition of extracellular matrix at the priming and activation stage of renal fibrosis in animal models by consulting PubMed and Cnki databases over the past 15 years. Curcumin exerts antifibrotic effect through reducing inflammation related factors (MCP-1, NF-*κ*B, TNF-*α*, IL-1*β*, COX-2, and cav-1) and inducing the expression of anti-inflammation factors (HO-1, M6PRBP1, and NEDD4) as well as targeting TGF-*β*/Smads, MAPK/ERK, and PPAR-*γ* pathways in animal models. As a food derived compound, curcumin is becoming a promising drug candidate for improving renal health.

## 1. Introduction

**Figure 1:**
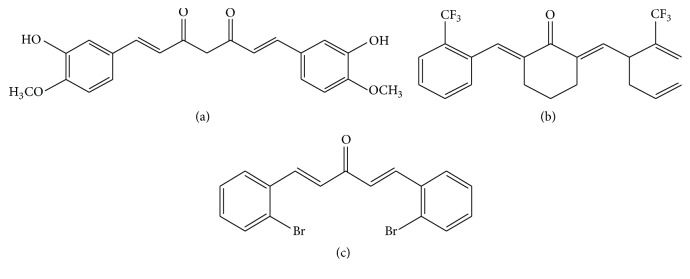
The chemical structure of curcumin (a), C66 (b), and B06 (c).

Curcumin, a polyphenol isolated from the Curcuma longa plant, is commonly known as turmeric in Asia (Figure 1) [1, 2]. As a traditional used herbal medicine and also a food spice in global cuisines, turmeric was reported to have extensively clinical applications in various kinds of diseases, such as asthma, fibrosis, diabetes, and abdominal pain [2–4]. Curcumin is one of active ingredients in turmeric and has been reported to attenuate the expression of apoptotic and chemokine genes in rat model of unilateral ureteral obstruction (UUO) in 2000 [5]. After that, a lot of researchers conducted various experiments to study the effects and mechanisms regarding curcumin as a potential source in the prevention and treatment of renal fibrosis. In addition, there is no toxicity concern rising when curcumin is taken at the recommended doses, which increased the potential of therapeutic agent of this compound. A number of reviews concerning the use of Chinese medicine for fibrosis have been recently published [4, 6–8]. This review intends to summarize the recent studies on curcumin in delaying advance of renal fibrosis through searching PubMed (https://www.pubmed.com/) and Cnki (https://www.cnki.com/) databases, which will provide additional evidence and also highlight the future research regarding curcumin in the management of kidney diseases.

Renal fibrosis is the principal process underlying the progression of chronic kidney disease (CKD) to end stage renal disease (ESRD). With a high prevalence of morbidity and mortality, CKD brings great pressure to patients and increases the burden on the society. In addition, currently there are no effective drugs to prevent the development of the ESRD. Characterized as glomerulosclerosis and tubular interstitial fibrosis, renal fibrosis is considered as a dynamic and converging process that consists of four overlapping phases: priming, activation, execution, and progression [9]. In the first stage, lasting inflammatory stimulation triggers the activation of renal tubular epithelial cells and the infiltration of inflammatory cells, including lymphocytes, monocytes/macrophages, dendritic cells, and mast cells [10]. During the activation and execution stages, profibrotic cytokines are released from injured tubular cells accompanying the activation of matrix-producing cells. It is accepted that the myofibroblasts are the main source of extracellular matrix (ECM), which derived from renal interstitial fibroblasts, bone marrow-derived fibrocytes, vascular pericytes, and endothelial and tubular cells by epithelial-to-mesenchymal transdifferentiation (EMT) [11]. The excessive deposition of ECM such as fibronectin and types I and III collagen contributes to the development of renal fibrosis. In the final stage, the renal structure and function gradually disappear with sustaining ECM deposition, which leads to the undesirable consequence of fibrosis.

## 2. Curcumin Is Involved in the Priming Stage of Renal Fibrosis

At the priming stage of renal fibrosis, inflammation initiates a fibrotic process [12]. Sustaining inflammatory stimulus triggers the activation of renal tubular epithelial cells and the infiltration of inflammatory cells, including lymphocytes, monocytes/macrophages, dendritic cells, and mast cells. Curcumin has been demonstrated to regulate multiple proinflammatory molecules and reduce recruitment of inflammatory macrophages [13–16] in various animal renal fibrosis models (Table 1).

Monocyte chemotactic protein-1 (MCP-1) is an important medium for monocyte/macrophage infiltration and a principle cytokine that may induce tubulointerstitial fibrosis (TIF) [17]. Macrophages are attracted to the site of injury by MCP-1 and its receptor CCR2. Blocking MCP-1/CCR2 pathway was shown to prevent kidney fibrosis through reducing recruitment of M1 inflammatory macrophages [18]. In the UUO rats models [5], curcumin treatment (0.5 mL of 30 mg/mL for 10 days, subcutaneous injection) significantly attenuated MCP-1mRNA overexpression in the obstructed kidney compared with that of control [5]. Further, curcumin treatment also decreased MCP-1 level in the factor-H-deficient mice (30 mg/kg for 5 weeks, intraperitoneal injection) [19] and lipopolysaccharide (LPS) stimulated mice (5 mg/kg for 3 days, intraperitoneal injection) [20].

Under the stimulation of inflammatory factors (interleukin (IL), tumor necrosis factor *α* (TNF-*α*)), nuclear factor-kappa B (NF-*κ*B) is activated and this activation further promotes the expression of transforming growth factor *β* (TGF-*β*) 1, intercellular adhesion molecule-1, and other fibrogenesis factors [33–35]. It has been demonstrated that curcumin treatment (100 mg/kg/day for 8 weeks, oral gavage) suppressed NF-*κ*B activation, prevented inhibitor of NF-*κ*B (I*κ*Ba) degradation, and decreased intercellular adhesion molecule-1 protein expression in streptozotocin- (STZ-) induced diabetic nephropathy rats [15], which was also reflected in LPS-induced kidney inflammation mice [20].

Proinflammatory cytokines, including TNF-*α* and IL-1, are involved in the development of chronic kidney disorders, including glomerulonephritis [36]. In 5/6 nephrectomy (5/6 Nx) rats, the high levels of TNF-*α* and IL-1*β* further triggered the production of cytosolic phospholipase A2 (cPLA2), calcium-independent intracellular PLA2 (iPLA2), and cyclooxygenase (COX) isoforms, which might contribute to inflammation [9]. Curcumin treatment (75 mg/kg, oral gavage) for 10 weeks significantly reduced the levels of the above-mentioned factors in 5/6 Nx rats [23]. In addition, administration of turmeric-based diet (5% w/w for 30 days) significantly decreased TNF-*α* mRNA expression in UUO rats [26]. C66 (0.2 mg/kg for 6 weeks, oral gavage), a novel curcumin derivative, has also been reported to reduce the production of TNF-*α*, IL-1*β*, COX-2, and NF-*κ*B in high glucose stimulated diabetic rats [37]. The above-mentioned results suggest that curcumin and its analogs may have strong ability of anti-inflammation in different renal rodent's diseases models.

Heme oxygenase-1 (HO-1) is the inducible isoform of the rate-limiting enzyme involved in the degradation of heme. It is a cytoprotective molecule that could restore renal function via resolving fibrosis factors [38, 39]. In anti-Thy1 glomerulonephritis rats, curcumin treatment (10 to 200 mg/kg, intraperitoneal injection) dose-dependently induced the expression of HO-1 in glomerular cells and antithymocyte serum nephritic rats [28]. The association of curcumin with HO-1 was further demonstrated by using zinc protoporphyrin (HO-1 inhibitor) in anti-Thy1 glomerulonephritis rats which resulted in loss of beneficial effects of curcumin on fibrosis and proteinuria [28]. In addition, curcumin treatment also increased HO-1 expression in the kidney of UUO rats [5]. The results also indicate that HO-1 agonists may offer new opportunity for renal diseases treatment.

Neural precursor cell expressed, developmentally downregulated 4 (NEDD4) family is closely related to inflammation, and mice lacking Nedd4 family interacting protein-1 developed severe inflammation in the skin and lung [40]. Mannose-6-phosphate receptor binding protein 1 (M6PRBP1) is involved in the metabolism of intracellular lipid. The levels of M6PRBP-1 and NEDD4 were reduced in response to LPS insulation [25]. Curcumin treatment (5 mg/kg for 3 days, intraperitoneal injection) increased renal M6PRBP1 and NEDD4 expression in LPS-induced kidney inflammation in Kunming mice [19]. Furthermore, the investigators also found that curcumin could inhibit the activation of mitogen-activated protein kinases (MAPK) and JNK-p38MAPK pathways by gene chip analysis [25], suggesting an important role in inflammation response [41, 42]. The results suggest that curcumin may have the effects on inflammatory cells proliferation, differentiation, and migration.

Caveolin-1 (cav-1) activation modulates innate immunity, inflammation, vascular permeability, and leukocyte migration [43]. Cav-1 binds to toll-like receptor 4 (TLR4), then mediates NF-*κ*B activation, and triggers the inflammatory response [44]. Curcumin treatment reduced cav-1 phosphorylation at Tyr14 and TLR4 activation in STZ-induced diabetic rats (100 mg/kg for 12 weeks, oral gavage) and high glucose stimulated mouse podocyte cell (curcumin, 1 to 10 *μ*M) [14].

In short, curcumin has been demonstrated to exhibit anti-inflammation properties in different kidney diseases models by reducing inflammatory molecules release (MCP-1, NF-*κ*B, TNF-*α*, IL-1*β*, COX-2, and cav-1) and inducing the expression of anti-inflammation factors (HO-1, M6PRBP1, and NEDD4), suggesting that it could play a contributing role in preventing the initiation of renal fibrosis.

## 3. Curcumin Is Also Actively Involved in Activation Stage of Renal Fibrosis

At the activation stage, profibrotic cytokines and factors are released from injured tubular cells, which stimulate the myofibroblasts to produce ECM. In addition, EMT further contributes to transdifferentiating endothelial and tubular cells to myofibroblasts [45]. An experiment performed by Sun et al. demonstrated that administration of curcumin (100 mg/kg for 12 weeks, oral gavage) prevented EMT through increasing the expression of epithelia cadherin, synaptopodin, and reducing expression of mesenchymal a-smooth muscle actin (*α*-SMA), fibroblast-specific protein 1 in the diabetic rats [21]. The possible mechanisms underlying these effects might be involved in suppressing the phosphorylation of cav-1 at Tyr14 and increasing stabilization of cav-1 and *β*-catenin. In addition, *β*-catenin favors EMT and renal fibrosis [46–48]. Curcumin inhibited high glucose induced dissociation of *β*-catenin from cav-1 and decreased active *β*-catenin expression [21]. In our group we also found that curcumin could inhibit the occurrence of EMT in renal tubular epithelial cells via regulating several sites of the TGF-*β*/Smads signal transduction pathway in UUO rats [49]. The inhibitory effect of curcumin on EMT was also demonstrated in cisplatin-induced renal fibrosis rats [31].

In addition, curcumin was proved to ameliorate EMT in TGF-*β*1 stimulated proximal tubular HK-2 cells through ERK and PPAR-*γ* dependent pathway [50]. Curcumin also exhibited similar effect in high glucose exposed NRK-52E kidney tubular epithelial cell through stimulating nuclear factor- (erythroid-derived 2-) like 2- (Nrf2-) mediated upregulation of HO-1 [51].

**Figure 2:**
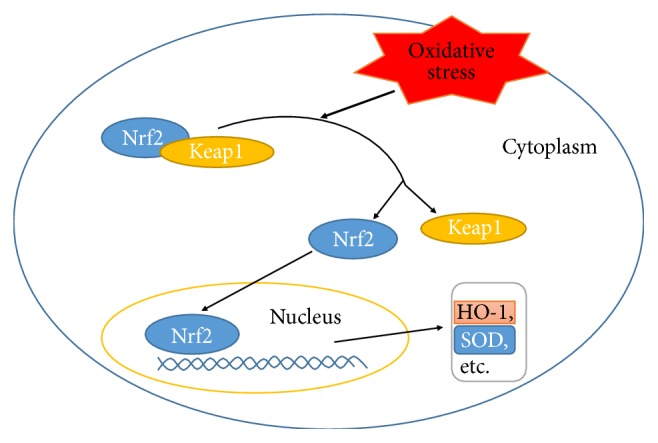
Nrf2 signaling pathway. Nrf2, as a transcription factor, resides within cytoplasm binding to the actin-associated Keap1 protein and is normally degraded. Upon oxidation stress, the association will be disrupted, resulting in the translocation of Nrf2 to nuclei and then increased expression of cytoprotective enzymes (HO-1, SOD, etc.).

The disequilibrium between oxidant and antioxidant system contributes to development of renal damage [52]. Curcumin (75 mg/kg for 16 days; oral gavage) was evidenced to inhibit the increase of inducible nitric oxide synthase (iNOS) expression in kidney in selenium-induced toxicity in Wistar rats [29]. The reducing level of iNOS facilitated removing oxidative/nitrosative stress. The protective effect of curcumin was more obvious in pretreatment group (administration of curcumin before selenium, 24 h) than simultaneous or posttreatment group (administration of curcumin after selenium treatment, 24 h). In rats with contrast-induced nephropathy [30], 5/6 Nx [24], and cisplatin-induced nephrotoxicity [31], curcumin treatment (60 and 120 mg/kg for 60 days [24]) increased antioxidant profiles (e.g., superoxide dismutase (SOD), enzymes catalase, glutathione reductase, glutathione peroxidase, and glutathione) and decreased oxidant profile (malondialdehyde) in the kidneys. Further, curcumin may exhibit renoprotective effect through Nrf2 translocation [31, 53], which beneficially contributes to ameliorating cisplatin-induced loss of tight junction proteins (claudin-2 and occludin) and adherens junction protein (E-cadherin and *β*-catenin) [31]. It is demonstrated that Nrf2 exerted cytoprotective effect through binding to antioxidant response elements [54, 55] (Figure 2). Interestingly, curcumin also exerted similar protective effects against oxidant stress induced renal damage between the pretreatment and posttreatment groups in 5/6 Nx rats. The inconsistency of the effects of curcumin administration approach between 5/6 Nx and selenium-induced rats may be owing to different renal diseases models, duration, and dosage. In rats with cyclosporine-induced nephrotoxicity, curcumin treatment (15 mg/kg for 21 days, subcutaneous injection) ameliorate renal injury by decreasing glutathione S-transferase immunoreactivity [32] which indicated that exogenous antioxidant curcumin might compensate the need of the renal cells to the endogenous glutathione antioxidant [42].

TGF-*β*/Smads signaling is considered as the most important pathway in the development of renal fibrosis [45]. TGF-*β*1 regulates the synthesis and degradation of ECM and induces the activities of fibrogenic cytokines that contributed to the development of fibrosis. TGF-*β*1 overexpression heavily favors fibrotic kidney disease [56]. Curcumin treatment inhibited TGF-*β*1mRNA expression [19]. And this inhibitory effect was mediated through reducing the phosphorylation of Smad2 and Smad3 [27]. In addition, pretreatment of curcumin resisted renal fibrosis by downregulating TGF-*β*1 receptor II in TGF-*β*1 stimulated NRK49F rat renal fibroblasts [57]. B06, one of the curcumin analogs, has also been proved to reduce the expression of collagen IV and fibronectin which further favored attenuating the accumulation of extracellular matrix and glomerular mesangial proliferation [58].

Furthermore, sphingosine 1-phosphate (S1P) activates TGF-*β* and contributed the renal fibrosis process [59]. However, the formation of S1P is catalyzed by sphingosine kinase 1 (SphK1) [60]. Huang et al. found that curcumin treatment (150 mg/kg for 12 weeks, oral gavage) significantly inhibited expression and activity of SphK1 and the production of S1P in STZ-induced diabetic rats [22].

In addition, our group found that curcumin treatment (20 *μ*M for 72 h) significantly decreased the expression of collagen I, *α*-SMA, and chemokine receptor 7 (CCR7), as well as TGF-*β*l secretion in human circulating fibrocytes [61]. The inhibitory effect of curcumin on the differentiation and migration of human circulating fibrocytes is likely through regulating the CCR7/CCL21 signaling pathway, in particular by reducing CCR7 expression.

The MAPK/ERK signaling pathway is also involved in the development of renal fibrosis [62, 63]. Pretreatment with curcumin blocked angiotensin II- (Ang-II-) induced profibrotic responses in renal tubular epithelial cells [64]. Ang-II exerted its fibrotic response and hypertension effect through TGF*β*1-MAPK/ERK pathway [65] and renin-angiotensin system. Pan et al. [37] further demonstrated that C66 prevented STZ-induced diabetic nephropathy through inhibition of MAPK mediated angiotensin converting enzyme (ACE) expression. Wang et al. [66] also found that the antifibrotic effect of C66 was exhibited through inhibition of JNK phosphorylation and p300/CBP-mediated histone acetylation. It is demonstrated that inhibition of histone deacetylase prevented renal interstitial fibroblasts activation and renal tubular cell apoptosis in a rat renal interstitial fibroblast line (NRK-49F) and in UUO mice models [67].

Emerging evidence suggests that peroxisome proliferator-activated receptor-*γ* (PPAR-*γ*) is implicated in cell cycle [68] and its agonists exert protective effect on glucose control, alleviating proteinuria and inhibiting tissue fibrosis [69]. In UUO mice, curcumin treatment (50 mg/kg and 100 mg/kg for 14 days, oral gavage) increased PPAR-*γ* expression and decreased phosphorylated Smad 2/3 [27]. This was also reflected in TGF-*β*1 stimulated proximal tubular epithelial cell HK-2 cells [50] and 5/6 Nx rats [16]. Since PPAR-*γ* is also associated with ACE [70, 71], it is also useful to probe the effect of curcumin on the ACE expression in renal diseases.

In summary, at activation stage of renal fibrosis, curcumin treatment inhibits EMT and rebuilds the oxidative-antioxidant balance. In addition, curcumin shows antifibrogenic properties by regulating TGF-*β* expression and blocking MAPK/ERK and PPAR-*γ* pathways.

## 4. Outlook and Conclusions

**Figure 3:**
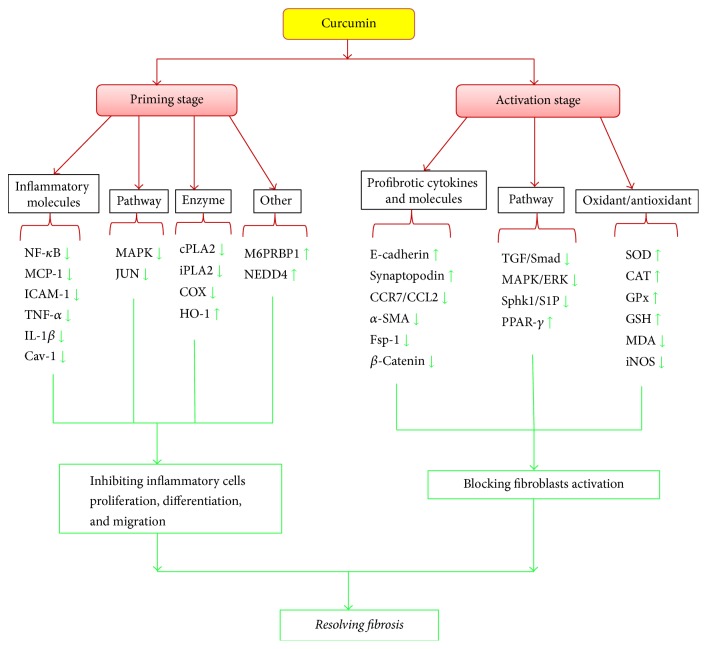
Curcumin plays a protective role at the priming and the activation stage of renal fibrosis. At the priming stage, curcumin reduces proinflammatory molecular activity and blocks inflammation associated signaling pathways. At the activation stage, curcumin inhibits the expression of renal fibrosis markers, rebuilds the redox balance, blocks MAPK/ERK pathway and TGF-*β*/Smads pathway, and increases PPAR-*γ* expression. NF-*κ*B, nuclear factor-kappa B; MCP-1, monocyte chemotactic protein 1; ICAM-1, intercellular adhesion molecule 1; TNF-*α*, tumor necrosis factor *α*; IL-1*β*, interleukin-1*β*; Cav-1, Caveolin-1; MAPK, mitogen-activated protein kinase; cPLA2, cytosolic phospholipase A2; iPLA2, calcium-independent intracellular PLA2; COX, cyclooxygenase; HO-1, heme oxygenase-1; CCR7, chemokine receptor 7; CCL21, chemokine ligand 21; *α*-SMA, *α* smooth muscle actin; Fsp-1, fibroblast-specific protein 1; TGF, transforming growth factor; Sphk1, sphingosine kinase 1; S1P, sphingosine 1-phosphate; PPAR-*γ*, peroxisome proliferators-activated receptor-*γ*; SOD, superoxide dismutase; CAT, catalase; GR, glutathione reductase; GPx, glutathione peroxidase; GSH, glutathione; MDA, malondialdehyde; iNOS, inducible nitric oxide synthase; NEDD4, neural precursor cell expressed, developmentally downregulated 4; M6PRBP1, mannose-6-phosphate receptor binding protein 1.

Curcumin has been demonstrated to be beneficially involved in resolving renal fibrosis at priming and activation stages through preventing inflammation initiation, rebuilding redox balance, inhibiting EMT, and resolving ECM excess deposition. These actions are mediated by reducing inflammation related factors (MCP-1, NF-*κ*B, TNF-*α*, IL-1*β*, COX-2, and cav-1) and inducing the expression of anti-inflammation factors (HO-1, M6PRBP1, and NEDD4) as well as targeting TGF-*β*/smads, MAPK/ERK, and PPAR-*γ* pathways in animal models (Figure 3). In addition, no data supports the notion that curcumin could restore renal injury during ESRD so far. Meanwhile, cautions must be excised that pretreatment and posttreatment may affect the effects of curcumin on renal fibrosis. Prospective studies are also needed to further elucidate the effects of curcumin in the development of renal fibrosis with in-depth understanding of this disease.

However, concerns are rising regarding the efficacy of curcumin in the management of renal fibrosis owing to its inherent low bioavailability. In most of the studies, curcumin was administrated by oral gavage. Further investigations are needed to explore the real active ingredients of curcumin after administration. Fortunately, some of curcumin derivatives with good bioavailability (such as C66 and B06) and new formulations of curcumin have been developed in recently years. However, the efficacy and safety of these new analogs and formulations remain largely unexplored. Taken together, as a food derived compound with golden-yellow fluorescence, curcumin may offer a new option in the treatment of renal fibrosis and also provide a new druggable chemical structure for chemists in designing new antifibrosis drug candidates.

## Figures and Tables

**Table 1:** The effects of curcumin in renal fibrosis models.

Animal model	Induction of renal injury (route and dose)	Route and dose	Course of treatment	Reference
Streptozotocin- (STZ-) induced diabetic rats	Intraperitoneal injection of STZ (60 mg/kg BW)	Oral gavage, 100 mg/kg (body weight) BW	12 weeks	[14]
STZ-induced diabetic rats		Oral gavage, 100 mg/kg BW	12 weeks	[21]
STZ-induced diabetic rats		Oral gavage, 150 mg/kg BW	12 weeks	[22]

STZ-induced diabetic rats.	Intraperitoneal injection of STZ (55 mg/kg BW in 20 mM citrate buffer, pH 4.5)	Oral gavage, 100 mg/kg BW	8 weeks	[15]

5/6 nephrectomy (5/6 Nx) rats	The upper and lower thirds of the left kidney and the whole right kidney were ligated and excised by surgery	Oral gavage, 75 mg/kg BW	8 weeks	[16]
5/6 Nx rats		Oral gavage, 75 mg/kg BW	10 weeks	[23]
5/6 Nx rats		Oral gavage, 60 and 120 mg/kg BW	7 days before and 60 days after 5/6 NX	[24]
5/6 Nx rats		Oral gavage, 120 mg/kg BW	30 days after 5/6 NX	[24]

Immune-complex-mediated glomerulonephritis	Intraperitoneal injection with 4 mg horse spleen apoferritin daily for 5 weeks	Intraperitoneal injection, 30 mg/kg BW	5 weeks	[19]

Lipopolysaccharide (LPS) induced renal inflammation mice	Intraperitoneal injection of LPS at the dose of 1 mg/kg BW or 5 mg/kg BW	Intraperitoneal injection, 1 mg/kg or 5 mg/kg BW	3 days	[20]

LPS induced kidney inflammation mice	Intraperitoneal injection with LPS (5 mg/kg body weight (BW))	Injection with curcumin (5 mg/kg) for 3 days before being injected with LPS	3 days	[25]

Unilateral ureteral obstruction (UUO) model of renal injury	The left ureter was ligated at two points with 3-0 silk	Turmeric-based diet with turmeric powder in a dose of 5% w/w	30 days	[26]

UUO male C57 mice	The left ureter was ligated with 4-0 silk	Gastrogavage, 50 and 100 mg/kg BW	2 weeks	[27]

Anti-Thy 1 glomerulonephritis model	Injected with the monoclonal antibody OX-7 at a dose of 2.2 mg/kg BW	Intraperitoneal injection, 10 to 200 mg/kg BW	Days 3 to 5	[28]

Selenium-induced oxidative stress rats	Intraperitoneal injection with selenium 15 *μ*M/kg BW	Oral gavage, 75 mg/kg BW	16 days	[29]

Contrast-induced nephropathy (CIN) rats	10 mg/kg furosemide IM + 10 mg/kg indomethacin IP + 10 mL/kg iomeprol IV was administered on the 5th day following 24 h dehydration under mild sevoflurane anesthesia	Oral gavage, 200 mg/kg BW	10 days	[30]

Cisplatin (CIS) induced nephrotoxicity	Intraperitoneal injection, 5 mg/kg CIS	Oral gavage, 200 mg/kg BW	Three doses (30 min before and 24 and 48 hours after CIS injection)	[31]

Cyclosporine-induced nephrotoxicity male rats	Subcutaneous injection of CsA dissolved in olive oil in a dose of 20 mg/kg BW daily	Oral gavage, 15 mg/kg BW	21 days	[32]
